# Efficient Anthocyanin Recovery from Black Bean Hulls Using Eutectic Mixtures: A Sustainable Approach for Natural Dye Development

**DOI:** 10.3390/foods13091374

**Published:** 2024-04-29

**Authors:** Mayara Kuasnei, Laís Benvenutti, David Fernando dos Santos, Sandra Regina Salvador Ferreira, Vânia Zanella Pinto, Acácio Antonio Ferreira Zielinski

**Affiliations:** 1 Department of Chemical Engineering and Food Engineering, Federal University of Santa Catarina, Florianópolis 88040-900, SC, Brazil; mayarakuasnei123@gmail.com (M.K.); labenvenutti@gmail.com (L.B.); s.ferreira@ufsc.br (S.R.S.F.); 2 Food Engineering, Federal University of Fronteira Sul, Laranjeiras do Sul 85301-970, PR, Brazil; davidfernandods@gmail.com

**Keywords:** anthocyanin stability, by-product valorization, cryoconcentration, eutectic solvents, indicators in packaging

## Abstract

There is a growing interest in exploring new natural sources of colorants. This study aimed to extract anthocyanins from broken black bean hulls (*Phaseolus vulgaris* L.) by modifying water with a eutectic mixture (choline chloride:citric acid (ChCl:Ca)). Ultrasound-assisted extraction (UAE) was employed and optimized in terms of temperature (30–70 °C), ultrasound power (150–450 W), and eutectic mixture concentration in water (1–9% (*w*/*v*)), resulting in an optimal condition of 66 °C, 420 W, and 8.2% (*w*/*v*), respectively. The main quantified anthocyanins were delphinidin-3-O-glycoside, petunidin-3-O-glycoside, and malvidin-3-O-glycoside. The half-life of the anthocyanins at 60 °C increased twelvefold in the eutectic mixture extract compared to the control, and when exposed to light, the half-life was 10 times longer, indicating greater resistance of anthocyanins in the extracted eutectic mixture. Additionally, the extracts were concentrated through centrifuge-assisted cryoconcentration, with the initial cycle almost double the extract value, making this result more favorable regarding green metrics. The first concentration cycle, which showed vibrant colors of anthocyanins, was selected to analyze the color change at different pH levels. In general, the technology that uses eutectic mixtures as water modifiers followed by cryoconcentration proved to be efficient for use as indicators in packaging, both in quantity and quality of anthocyanins.

## 1. Introduction

The common bean (*Phaseolus vulgaris* L.) has long been fundamental in human nutrition [1,2]. Bean seeds are an excellent source of proteins, as well as carbohydrates, minerals, vitamins, and bioactive compounds; their consumption can reduce the incidence of some diseases [3,4]. This legume is widely consumed worldwide, and Brazil stands out as the second-largest global producer of beans, with an annual production of 2.9 million tons in 2019 [5]. However, grain losses during harvesting, processing, and storage are mostly due to mechanical damage, which causes grain breakages and reduces its commercial value [6,7]. Due to the high amount of broken beans in its processing chain (from 6 to 13%), the valorization and industrial use of this waste has been investigated [8,9].

In this context, the recovery of anthocyanins can be a good alternative, supported by an anthocyanin recovery of 1.21 to 4.83 mg of cyanidin-3-glucoside equivalents (C3GE) per gram [8,9]. Anthocyanins have antimicrobial and antioxidant properties, enhancing food shelf files and offering natural color indicators in food packaging. Their use as colorimetric indicators based on pH changes is widely studied due to their simplicity and non-invasiveness, being easily detectable visually by retailers and consumers [10,11].

For them to be used as indicators on the packaging or natural colorants in foods, cosmetics, and pharmaceutical products, it is necessary to extract these substances from the plant matrix [8,12]. Ultrasound-assisted extraction (UAE) stands out as an environmentally friendly and economically viable alternative method, with a remarkable ability to extract bioactive compounds [13,14]. This process involves the propagation of sound waves in a specific medium, which generates significant shear forces, leading to the formation and implosion of bubbles, known as cavitation. UAE improves mass transfer, reduces extraction time, provides high yields, and is easy to operate [13,15].

Based on the green extraction strategies, deep eutectic solvents (DES) or eutectic mixtures have shown high potential to replace conventional organic solvents [12]. These solvents can increase the extraction yield and improve the shelf-life of bioactive-rich extracts, protecting against adverse conditions (e.g., temperature, light, and storage) [9,16]. The main disadvantage of these solvents is their viscosity, which can harm extraction efficiency. However, this obstacle can be overcome when eutectic mixtures are used as a water modifier to carry out the extractions [17]. Eutectic mixtures added in water can be an alternative green solvent since water is a non-hazardous substance and is considered the greenest solvent [18]; and depending on their composition, eutectic mixtures can be environmentally friendly and non-toxic [19,20].

To preserve the quality of aqueous extracts and make them more practical for transportation and use, it is desirable to concentrate the recovered bioactive compounds. Cryoconcentration is a technique used to concentrate liquids and recover heat-sensitive compounds [21,22]. This approach involves removing water through the formation of ice crystals during the cooling process. These crystals are subsequently separated from the concentrated, unfrozen fluid [23]. Cryoconcentration offers several advantages compared to other concentration methods, especially due to the low temperatures used, resulting in high-quality products [21,24]. Research has demonstrated that high temperatures can cause hydrolysis of the glycosidic bond in anthocyanins, with this hydrolysis considered the main cause of the loss of color in these compounds [25,26]. Therefore, the use of alternative low-temperature methods to concentrate extracts rich in anthocyanins gains relevance.

Thereby, the aims of this study were (i) to enhance the extraction of anthocyanins from broken black bean hulls using an ultrasound-assisted extraction technique, utilizing a water-based eutectic mixture as the solvent; (ii) to assess the stability of the resulting anthocyanin-rich extract in an aqueous eutectic mixture medium; (iii) explore the potential of cryoconcentration to obtain high-quality extracts rich in anthocyanins, aiming to preserve their color for possible use as indicators in packaging.

## 2. Materials and Methods

### 2.1. Sample and Chemicals

Broken black beans (*Phaseolus vulgaris* L.) were donated from bean processing industries from Ponta Grossa City, Parana (Brazil), in 2019. The chemicals Folin–Ciocalteu, Trolox (6-hydroxy-2,5,7,8-tetremetricroman-2-carboxylic acid), DPPH (1,1-diphenyl-2-picryl-hydrazyl), ABTS (2,2′-azinobis (3-ethylbenzothiazoline-6-sulfonic acid)) were purchased from Sigma-Aldrich (Steinheim, Germany). Choline chloride and citric acid were also purchased from Sigma-Aldrich (Steinheim, Germany). All other reagents used for the analysis were of analytical grade.

### 2.2. Sample Preparation

Broken black beans were selected and hydrated (1:4, *w*/*v*) at 4 °C for 2 h, followed by manual separation of hulls and cotyledons. Afterward, the hulls were dried in an oven with forced air circulation (DeLeo, Porto Alegre, Brazil) at 60 °C for 17 h and crushed in a Willey knife mill (DeLeo, Porto Alegre, Brazil). The particle size of the hull powder was standardized by a set of sieves (mean diameter 0.48 ± 0.01 mm), with openings from 20 to 32 mesh, from the Tyler series (W.S. Tyler, Wheeling, WV, USA).

The eutectic mixture was formed by choline chloride:citric acid (ChCl:Ca) in a molar ratio of 1:1, which was prepared by the mixture of the starting components under heat at 80 °C and continuous stirring using a Dubnoff bath (Ethik technology, model 304-TPA, São Paulo, Brazil) until a homogenous liquid is obtained. The selection of the eutectic mixture used was based on previous studies [9] due to the high extractability of anthocyanins.

### 2.3. Optimization of the Ultrasound-Assisted Extractions (UAE)

The conditions for the UAE were optimized according to a Box–Behnken design on three levels, performed randomly. The UAE assays were conducted using 0.5 g of dry powder samples and 15 mL of solvent (aqueous solution of eutectic mixture), placed in an extraction jacketed cell using an ultrasound probe (Eco-sonics, São Paulo, Brazil), with 20 kHz frequency. The process was monitored for 10 min (time defined previously by kinetic). The process variables, evaluated in three levels, were: temperature, at 30, 50, and 70 °C; ultrasound power, 150, 300, and 450 W; and eutectic mixture concentration as water modifier of 1, 5, and 9%. The recovered extracts were centrifuged at 4515× *g* (Quimis, Q222T, Diadema, Brazil) for 10 min, and stored in amber flasks until further analysis.

The dependent variables were total monomeric anthocyanins, total phenolic content, and antioxidant activity measured by DPPH and ABTS (Section 2.4). The independent variables were temperature, ultrasound power, and eutectic mixture concentration added to water. The experimental dataset was assessed using the response surface methodology (RSM) and multiple regression analysis. The mathematical models developed were derived from a generalized second-order polynomial model (Equation (1)).

(1) $$Y_{n}\left(x\right)=\beta_{0}+\sum_{i=1}^{3}\beta_{i}x_{i}+\sum_{i=1}^{3}\beta_{ii}x_{i}^{2}+\sum_{i<j=1}^{3}\beta_{ij}x_{i}\;x_{j}$$

In the equation, the predicted response (*Y*) is determined by the regression coefficients *β*_0_, *β_i_*, *β_ii_*, and *β_ij_*, representing the intercept, linear, quadratic, and interaction terms, respectively. The independent variables, denoted as *x_i_* and *x_j_*, correspond to the temperature (*x*_1_), ultrasound power (*x*_2_), and concentration of eutectic mixture in water (*x*_3_).

Analysis of variance (ANOVA) was used to evaluate the statistical significance of the model parameters. Parameters that did not demonstrate statistical significance were removed from the mathematical model, resulting in a reconfiguration of the data that included only significant parameters (*p* < 0.05). The quality parameters of the models were evaluated by *p*-value, regression coefficient (R^2^), and its respective adjusted R^2^. After this evaluation, the response surfaces were built.

To obtain a simultaneous optimal condition, maximizing the content of TMA, TPC, and antioxidant activity, the desirability function was used [27]. Lastly, external validation of the model was carried out in the optimal condition determined to verify the adequacy of the model.

### 2.4. Characterization of Anthocyanin-Rich Extracts

#### 2.4.1. Total Monomeric Anthocyanin (TMA)

The methodology used to determine the content of total monomeric anthocyanins (TMA) in the extracts consisted of analysis using the differential pH method [28]. Absorbances (520 nm and 700 nm) were measured using a microplate reader (Multileader Infinite M200 TECAN, Zürich, Switzerland). The results were expressed in mg of cyanidin-3-O-glucoside per gram of dry sample (mg g^−1^), calculated according to Equations (2) and (3).

(2) $$A={\left(A_{520}-A_{700}\right)}_{pH\;1.0}-{\left(A_{520}-A_{700}\right)}_{pH\;4.5}$$

(3) $$TMA=\frac{\left(A\times MW\times DF\times 10^{3}\right)}{\varepsilon \times 1}$$

where A_520_ and A_700_ represent the absorbance readings, and A is the calculated absorbance. MW denotes the molecular weight of the anthocyanin standard (cyanidin-3-O-glucoside: 449.2 g mol^−1^), F is the dilution factor, and ε is the molar absorptivity of cyanidin (26,900).

#### 2.4.2. Total Phenolic Content (TPC)

The total phenolic content (TPC) was assessed using the Folin–Ciocalteu reagent, modified for use with a microplate reader following the method by Singleton and Rossi [29]. The TPC concentration was calculated using a calibration curve (TPC = 0.0025 × A + 0.039, where A represents the absorbance, R^2^ = 0.99) of gallic acid. The results were expressed as milligrams of gallic acid equivalent (GAE) per gram of dry matter (mg GAE g^−1^).

#### 2.4.3. In Vitro Antioxidant Activity (AA)

The in vitro antioxidant activity was assessed using the DPPH and ABTS free radical scavenging methods. The DPPH assay was performed according to [30]. For this, 50 μL of diluted extracts and 250 μL of DPPH (125 μmol L^−1^ in methanol) were added to the microplates.

The evaluation of in vitro antioxidant activity was conducted using the DPPH and ABTS free radical scavenging methods. The DPPH assay was performed as described by [30]. For this, 50 μL of diluted extracts were combined with 250 μL of DPPH (125 μmol L^−1^ in methanol) in microplates. After 30 min of reaction, the absorbance was measured at 517 nm using a microplate reader (Multileader Infinite M200 TECAN, Zürich, Switzerland).

For the ABTS assay, the modified microplate method described by Re et al. [31] was employed. In the microplate, 20 µL of the diluted samples were mixed with 280 µL of the ABTS radical solution, and the absorbance was then measured at 734 nm. The scavenging activity of both DPPH and ABTS radicals was calculated using Equation (4).

(4) $$Antioxidant\;Activity\left(\%\;inhibition\right)=\left[1-\left(\frac{Abs\;sample}{Abs\;white}\right)\right]\times 100$$

The antioxidant activity for both methods was determined through triplicate assays and calculated using a calibration curve (DPPH = 0.339 × A + 0.099, R^2^ = 0.99, and ABTS = 0.137 × A + 0.3765, R^2^ = 0.99) of Trolox, with results expressed in μmol of Trolox equivalent per g of dry sample (μg TE g^−1^).

#### 2.4.4. Identification and Quantification of Individual Anthocyanins

The identification and quantification of anthocyanins in the samples were carried out according to previous studies [9], with slight modifications, using a liquid chromatography (Shimadzu, Kyoto, Japan) equipped with a diode array detector (SPD-M20A) and a mass spectrometer with electrospray ionization (LCMS-2020). Separation was achieved by injecting 10 μL of the sample onto a C18 column (NST, Santos, Brazil) measuring 4.6 mm × 250 mm, with a particle size of 5.0 μm, operating at 40 °C. The mobile phase consisted of solvent A (water with 0.1% formic acid in, *v*/*v*) and solvent B (methanol acidified with 0.1% formic acid), with a flow rate of 1.2 mL min^−1^. The gradient elution program was set as follows: 0–16 min, 14−55% B; 16–27 min, 55–100% B; 27–30 min, 100–14% B; 30–32 min, 14% B. The rotary spray spectrum interface was maintained at a temperature of 350 °C, with a nebulizer gas flow rate of 1.5 L/min, a heat block temperature of 200 °C, and a drying gas flow rate of 15 L/min. The interface voltage was set at 4.5 kV, and the RF-beam voltage at 60 V. Anthocyanin identification was achieved by confirming the masses (*m*/*z*) in the mass spectrometer. All analyses were performed in triplicate.

### 2.5. Stability of Anthocyanin-Rich Extracts

#### 2.5.1. Impact of Temperature on Anthocyanin Degradation

The thermostability of anthocyanin-rich extracts was determined according to the method proposed by Peron et al. [32]. The anthocyanin-rich extracts were placed in a thermostatic bath (Fisatom, model 550, São Paulo, Brazil) at different temperatures (60–90 ° C) at different times (15–300 min). For comparison, an extraction using only water was also carried out under the same conditions. After this process, the constant degradation rate (k) for the kinetic experiments for each temperature was calculated by a first-order model, as described by Equation (5).

(5) $$C_{A}=C_{A0}\cdot e^{-k\cdot t}$$

where $C_{A}$ is the anthocyanins concentration at time t, (mg g^−1^);$\;C_{A0}$ is the anthocyanins concentration at time zero t_0_ (mg g^−1^); t is the experimental time (s) and k is the constant degradation rate (min^−1^).

From the data of kinetics degradation, the half-life time (t_1⁄2_) (Equation (6)), the decimal reduction time (D value) (Equation (7)), and the temperature coefficient (Q_10_) (Equation (8)) were determined.

(6) $$t_{\frac{1}{2}}=\frac{\ln\left(0.5\right)}{k}\;$$

(7) $$D=\frac{\ln\left(10\right)}{k}$$

(8) $$Q_{10}={\left(\frac{\kappa_{T_{2}}}{\kappa_{T_{1}}}\right)}^{\frac{10}{T_{2}-T_{1}}}\;$$

where${\;t}_{\frac{1}{2}}$ = half-life time, D = decimal reduction time, $\kappa_{T_{1}}$ = kinetic constant of degradation concerning temperature *T*_1_; $\kappa_{T_{2}}$ = kinetic constant of degradation in relation to the temperature *T*_2_; *T*_1_ = temperature (°C) and *T*_2_ = temperature (°C).

##### Thermodynamics of the Degradative Process

The activation energy ($E_{a}$) was determined using the Arrhenius equation (Equation (9)):

(9) $$\ln k=\ln A-\frac{E_{a}}{R.T}$$

which, A = frequency factor (s^−1^);$\;E_{a}$ = activation energy of the degradation reaction (kJ mol^−1^), *T* = temperature (K) and *R* = ideal gas constant in (J mol^−1^ K^−1^).

The variation of the enthalpy of activation (ΔH) was defined by Equation (10):

(10) $$∆H=E_{a}-R.T$$

where ΔH = enthalpy change (kJ/mol); $E_{a}$ = activation energy of the degradation reaction (kJ mol^−1^); *R* = ideal gas constant (kJ mol^−1^ K^−1^) and *T* = temperature (K).

Gibbs free energy (ΔG) was determined as expressed in Equation (11):

(11) $$∆G=-R.T\ln\left(\frac{\kappa_{d.h}}{\kappa_{B.}T}\right)$$

where ΔG = variation of Gibbs free energy (kJ mol^−1^); *R* = ideal gas constant in (J mol^−1^ K^−1^); *T* = temperature (K); *k* = thermal degradation constant in (s^−1^); *h* = Planck’s constant, (6.6262 × 10^−34^ J s) and *kB* = Boltzmann’s constant (1.3806 × 10^−23^ J K^−1^).

Activation entropy (ΔS) was defined by Equation (12):

(12) $$∆S=\frac{∆H-∆G}{T}\;$$

where ΔS = entropy change (kJ mol^−1^ K^−1^).

#### 2.5.2. Effect of Light on Anthocyanin Degradation

The light effect was determined at room temperature (20 ± 2 °C) following the methods described by Dai et al. [16]. Anthocyanin extracts extracted with a eutectic mixture or with water (control) were exposed to direct light from a white fluorescent lamp (20 W) in a closed wooden chamber (435 × 435 mm). The retention of total monomeric anthocyanins (2.4.1) was determined at times of 0, 3, 6, 9, 12, 24, 36, 48, 60, and 72 h. The constant degradation rate of anthocyanins (k) and their half-life (t_1/2_) were calculated according to Equations (5) and (6).

#### 2.5.3. Block Cryoconcentration Assisted by Centrifugation

The cryoconcentration of the anthocyanin-rich extract was performed based on the methodology proposed by Petzold et al. [33]. In centrifuge tubes, 15 mL of the anthocyanin-rich extracts were frozen in a static freezer at –20 ± 2 °C for 12 h. The frozen samples were centrifuged (Quimis, model Q222T, Apparatus Scientific LTDA, São Paulo, Brazil) for 15 min at 4515× *g*, to provide the solute separation from the frozen samples, obtaining two fractions: concentrate and ice. Then, the fractions were evaluated in terms of anthocyanin content. The concentrated fraction was divided into 15 mL centrifuge tubes and frozen again at −20 ± 2 °C for 12 h, and the same process was repeated for 2 more cycles. The percentage of concentration (*Pc*) and the concentration efficiency (*n*) were calculated according to Equations (13) and (14), respectively

(13) $$Pc\left(\%\right)=\frac{W_{i}^{0}-W_{i}^{f}}{W_{i}^{0}}\cdot 100$$

where *Pc* = percentage of concentrate (%), $w_{i}^{0}$ e and $w_{i}^{f}$ = initial and final masses of the frozen fraction (g), respectively.

(14) $$n\left(\%\right)=\frac{C_{C}-C_{i}}{C_{C}}\cdot 100$$

where $C_{C}$ and $C_{i}$ = concentrations of total manometric anthocyanins in the concentrated solution and in the frozen fraction, respectively.

The results were validated according to the theoretical mass balance of each cryoconcentration cycle, according to Equations (15) and (16).

(15) $$W_{e}=\frac{m_{ice}}{m_{initial}}$$

(16) $$W_{p}=\frac{C_{C}-C_{0}}{C_{C}-C_{f}}$$

where $C_{0}$ = initial solids concentration; $C_{f}$ = final solids concentration; *W_e_* = experimental value and $W_{p}$ = predicted value.

The comparison between the experimental data (*W_e_*) and the predicted values (*W_p_*) for each cryoconcentration cycle, with N experimental points, was carried out by calculating the root mean square (*RMS*) (Equation (17)):

(17) $$RMS\left(\%\right)=100\sqrt{\frac{\sum {\left[\left(W_{e}-W_{p}\right)/W_{e}\right]}^{2}}{N}}$$

### 2.6. Green Metrics

Green metric tools as the environmental factor (E-factor), Green Certified, and EcoScale were used to measure the sustainability of the extraction and cryoconcentration process (1. Extraction; 2. Extraction + Cryoconcentration 1; 3. Extraction + Cryoconcentration 1 + Cryoconcentration 2; 4. Extraction + Cryoconcentration 1 + Cryoconcentration 2 + Cryoconcentration 3) to obtain anthocyanin-rich extracts from broken black bean hulls. The E-factor was calculated according to Wang et al. [34] (Equation (18)):

(18) $$E-factor=\frac{total\;mass\;of\;waste}{mass\;of\;the\;desired\;product}$$

Alternative approaches incorporate Penalty Points (PP) to assess the degree of environmental safety in a process, taking into account various parameters with assigned PP values to reduce the overall score from 100%. The Green Certificate methodology considers parameters such as solvent quantity, environmental hazard, energy consumption, and waste generation, which were calculated as described by Espino et al. [35]. On the other hand, the EcoScale approach integrates these parameters with factors such as process yield, safety, economic viability, and overall environmental impact of the production process [36].

### 2.7. UV-Vis Spectroscopy at Different pHs

The UV–vis spectra of the concentrated anthocyanin extracts were measured using a UV–vis spectrophotometer (EPOCH, BioTek, Ribeirão Preto, São Paulo, Brazil) in the range of 300 to 700 nm. Buffer solutions were prepared in different pH ranges (1–12). The anthocyanin concentrate from the first cycle was diluted in these buffer solutions, where 100 uL of the concentrate was diluted to 5 mL at the corresponding pHs, providing a wide range of different pHs, ranging from 1 to 12.

### 2.8. Statistical Analysis

All results were presented as mean ± standard deviation. The statistical analyses were performed using Statistica v. 13.5 software (TIBCO Software Inc., Palo Alto, CA, USA).

## 3. Results and Discussion

### 3.1. UAE Optimization

The optimization of process conditions, which include factors such as temperature, solvent concentration, solid-liquid ratio, and particle size of the plant matrix, is essential to develop an extraction method effective in recovering bioactive compounds [37]. In this context, the approach of the three-level Box–Behnken design, combined with the Response Surface Methodology (RSM), is suitable for optimizing the extraction process, making it economically efficient [38,39]. The results of the tests using this approach for the extraction conditions are summarized in Table 1. During the tests, the TMA values varied from 1.6 to 4.1 mg.g^−1^, the TPC content varied from 37 to 112 mg GAE g^−1^, and in vitro antioxidant activity, measured by the DPPH method, ranged from 248 to 434 μmol TE g^−1^, while by the ABTS method, it ranged from 373 to 966 μmol TE g^−1^.

The mathematical models from the experimental design results (Table 1) significantly (*p* < 0.05) described the relationship between the process variables evaluated in the UAE. Furthermore, the models did not show a lack of fit (*p* > 0.05), except for TMA (*p =* 0.01). However, due to the high R^2^_ajd_ > 0.92 for the TMA, the mathematical model is considered valid even when the *p*-value lack of fit is less than 0.05 [40].

**Figure 1:**
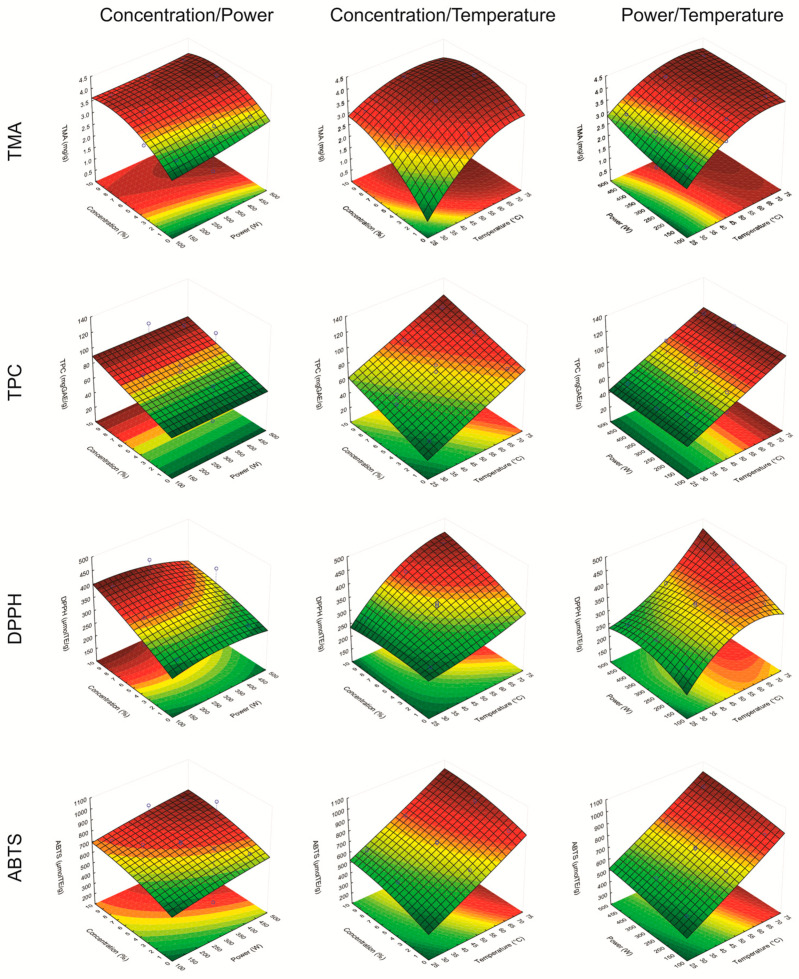
Response surface for total monomeric anthocyanins (TMA), phenolic total compounds (TPC), and antioxidant activity (DPPH and ABTS), obtained by the UAE method.

In all proposed models, the linear effects of temperature (*x*_1_), ultrasound power (*x*_2_), and concentration of the eutectic mixture as water modifier (*x*_3_) had a positive and significant impact on extraction performance for all dependent variables studied. This is evidenced by the response surface plots (Figure 1). The positive linear effect of *x*_1_ indicates that increasing temperature resulted in improvements in TMA, TPC, DPPH, and ABTS levels.

The temperature provided an increase in the desorption and solubility of the solute in the solvent, in addition to improving the diffusivity of the solvent in the vegetable matrix [41]. However, for TMA and DPPH, the negative quadratic effect of temperature (*x*_11_) indicated that a limit of temperature must be determined to obtain a maximum yield. This fact is related to the degradation of thermosensitive compounds, such as anthocyanins [42,43]. Similarly, the ultrasound power (*x*_2_) presented positive linear effects for all dependent variables and negative quadratic effects for TMA and DPPH, respectively. According to Kumar et al. [41], the cavitation bubble size is proportional to ultrasound power, which intensifies the explosions and, consequently, the fragmentation and pore formation of the vegetal matrix, increasing the solvent diffusivity and the extraction yield. However, the increase in the intensity of the explosions can lead to the degradation of anthocyanins due to the increase in temperature provided by high-speed collisions between the particles [15].

Overall, an increase in the levels of TMA, DPPH, and ABTS was observed at the higher eutectic mixture concentration added in the water as a modifier. According to Guo et al. [44], the mixture of water with other solvents is widely applied in extractions, adjusting the solvent polarity and improving the extraction efficiency. Therefore, the use of eutectic mixture (ChCl:Ca), as a water modifier was suitable for anthocyanin recovery by UAE. The high extraction capacity of the eutectic ChCl:Ca mixture may be related to its molecular interactions, mainly hydrogen bonds, between the DES component with target molecules and the water [45,46].

From the mathematical models that describe the UAE process, a multi-response optimization using the desirability function suggested an optimum extraction at a temperature of 66 °C, ultrasound power of 420 W, and eutectic mixture concentration of 8.2% of ChCl:Ca as water modifier. The goal achieved with optimization was more than 91% (d = 0.9187). All optimization points were externally validated, providing values within the predicted results for a 95% confidence level: TMA (mgC3GE·.g^−1^) observed: 3.58; predicted: 4.41, TPC (mg·GAE·g^−1^) observed: 75.48; predicted: 102.59, DPPH (μmolTEg^−1^) observed: 406.45; predicted: 409.12, ABTS (μmolTEg^−1^) observed: 891.63; predicted: 949.01. Therefore, for all variables evaluated, the models can be used to predict the response values.

### 3.2. Individual Anthocyanins in Optimized Extract

In the optimized anthocyanin-rich extract, the main individual anthocyanins identified by their respective ions were delphinidin-3-O-glycoside [M+] 465.1, petunidin-3-O-glycoside [M+] 479.12, and malvidin-3-O-glycoside [M+] 493.13, which corresponded at the same mass determined by Mojica et al. [47]. The authors also concluded that these anthocyanins are the main ones found in black beans.

In the present study, delphinidin-3-O-glycoside represented the largest proportion of 42.7% of the total, followed by petunidin-3-O-glycoside with 36.3% and malvidin-3-O-glycoside with 20.8% of the total. Although anthocyanins have a similar structure, individual anthocyanins exert different influences on the bioactivity of the extract. Malvidin corroborates with anti-obesity potential since it can promote osteogenesis of stem cells [48]. Delphinidin can inhibit adipogenesis in mesenchymal stem cells, presenting similar anti-obesity drugs, liraglutide. According to Cvorovic et al. [49], delphinidin can also be useful to decrease the risk of colon cancer cell metastases. Furthermore, delphinidin and cyanidin are the main monomeric anthocyanins related to anti-diabetic potential [50,51]. Therefore, the obtained extract can be a potential health-beneficial ingredient for food and pharmaceutical formulations.

### 3.3. Thermo and Photostability of Anthocyanin-Rich Extracts

The evaluation of thermal stability revealed a constant rate of anthocyanin degradation (*k*), which increased with temperature. In the anthocyanin-rich extract obtained using 8.2% of the eutectic mixture as a water modifier, the *k* value varied from 0.0004 to 0.005 min^−1^. In contrast, in the control extract, which used only water as a solvent, *k* varied from 0.005 at 0.013 min^−1^. This means there was less anthocyanin degradation during heating (60–90 °C) in the extract recovered with the eutectic mixture added in water, compared to the extract that used only water as solvent. This result suggests that the type of solvent affects the rate of anthocyanin degradation during heating.

The time required to halve the amount of anthocyanins (t_1/2_) was significantly longer for the extract obtained with the eutectic mixture as a water modifier compared to the control extracts (Table 2). For example, at 60 °C, the time was approximately 12 times longer for the extract with the eutectic mixture compared to the extract containing only water. This reinforces the idea that even at low concentrations, eutectic mixtures exert a protective effect on anthocyanin molecules during heating, which is also reflected in decimal reduction times.

The higher anthocyanin stability in the eutectic mixture medium is due to extensive hydrogen bonds between anthocyanins and eutectic mixture components, which reduces the molecule mobility and susceptibility to degradative reactions [16,52]. According to Dai et al. [53], *Catharanthus roseus* petal extracts recovered with eutectic solutions (lactic acid–glucose) also showed higher stabilities compared to other solvents (ethanol acidified with formic acid), justifying the stability efficiency due to hydrogen bonds between anthocyanins and eutectic mixtures components.

**Figure 2:**
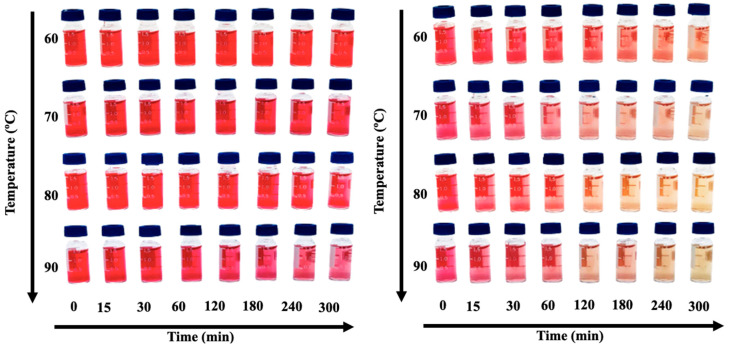
Effect of heating at temperatures of 60, 70, 80, and 90 °C overtime on the stability of the aqueous extract of anthocyanins, using 8.2% of the eutectic mixture (**left**) as water modifier and water as control (**right**).

Furthermore, the Q_10_ values calculated in our study showed significant differences in thermal sensitivity between the anthocyanins extracted with water modified by DES and those extracted with water alone as the solvent. These differences reflect distinct thermal degradation constants for each type of analyzed extract. In addition to the calculated parameters, the formation of brown-colored pigments was also observed in extracts obtained using water alone, indicating the degradation of anthocyanins. Even in small quantities, the eutectic mixture demonstrated the ability to preserve anthocyanins (Figure 2).

The activation energy ($E_{a}$) values for the extract samples with the eutectic mixture and with water were 87.38 kJ mol^−1^ (R^2^ = 0.9815) and 30.05 kJ mol^−1^ (R^2^ = 0.9555), respectively. The results show that anthocyanin-rich extracts obtained by water modified by eutectic mixture require more energy to activate the thermal degradation reaction, confirming the slower degradation process [32].

The activation enthalpy (ΔH) was positive for both extract samples, indicating an endothermic state, with higher degradation rates with increasing temperature. The reduced ΔH for the water extract, compared to the eutectic mixture extract (8.2% ChCl:Ca) indicated a lower energy barrier to break the bonds. Then, it is easier to degrade anthocyanins from water extract compared to eutectic mixture extract [54].

Gibbs free energy (∆G) represents the spontaneity of the reaction. ∆G values were consistently positive for both extracts across all temperatures (Table 2), according to Peron et al. [32] and Georgieva et al. [55] implies that anthocyanin degradation was not spontaneous since the temperatures in the assays accelerated the degradation of anthocyanins. The activation entropy (ΔS), which measures changes in molecular disorder, was negative for both extracts, indicating that anthocyanins undergo reorganization during the degradation process [55,56].

The light effect was also evaluated; the degradation rate (k) was lower for the anthocyanin-rich extract (obtained using 8.2% of the eutectic mixture as water modifier), (0.004 day^−1^) compared to the control (obtained using only water as solvent extract) (0.035 day^−1^), with a half-life 10 times higher for eutectic mixture extracts. The stability of the control extracts was reduced by approximately 70% in the first 24 h, changing from reddish pink to light pink, while the eutectic mixture extracts showed a pink-reddish color from the beginning to the end of the analysis. Overall, the anthocyanin-rich extracts obtained by water modified by eutectic mixture presented thermostability and photostability for different process conditions.

### 3.4. Cryoconcentration of Anthocyanin-Rich Extracts

**Figure 3:**
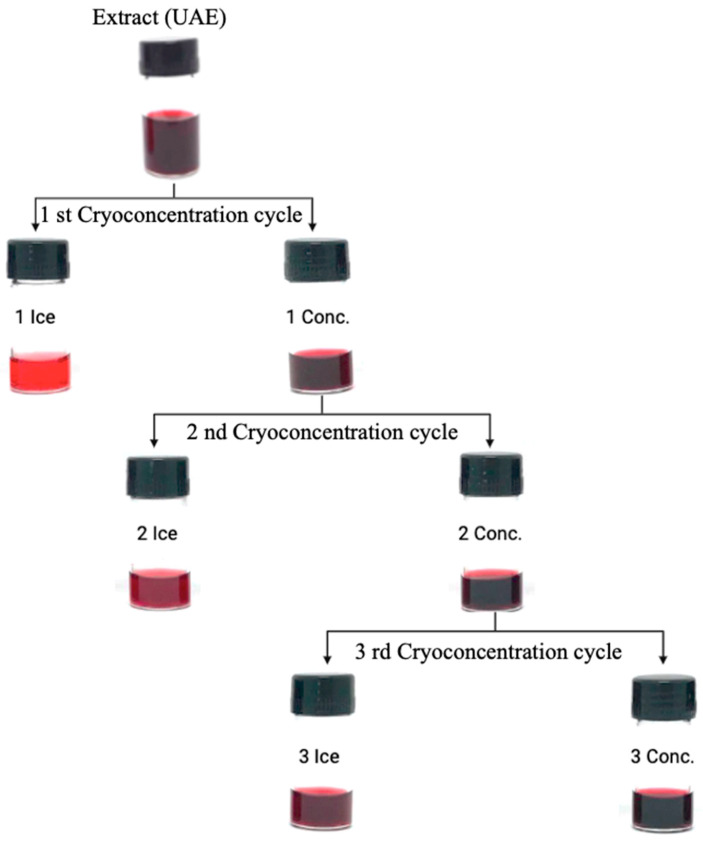
Cryoconcentration Process: Enhanced Experimental Approach.

Based on the growing demand for non-heating techniques, cryoconcentration has emerged as a viable alternative for concentrating anthocyanin-rich extracts. The concentration outcomes are outlined in Table 3. Notably, as the number of cryoconcentration cycles rises, so do the TMA values in the extracted sample. For instance, in the third cycle, the initial concentration surged by approximately 2.6 times the initial value (Figure 3).

Throughout the cycles, there was a steady rise in the percentage of concentrate, reaching 85% (as shown in Table 3). However, in terms of efficiency, an inverse trend was observed in the percentage of concentrate. Petzold et al. [57] suggest that this decline in efficiency could be linked to the escalating concentration of anthocyanins during the cycles. Consequently, an increase in the viscosity of the extract occurred, making water removal more challenging.

The validation of cryoconcentration, as presented in Table 3, involved a comparison between experimental ice mass relationships (We) and predicted values (Wp). The Root Mean Square (RMS) values, ranging from 1.72 to 5.10% across the cryoconcentration steps studied, serve as indicators of the accuracy of the predictive model. These RMS values are consistent with findings by Petzold et al. [33], which suggest that values below 25% are acceptable for validating cryoconcentration processes. This validation process is crucial for establishing the reliability and robustness of the cryoconcentration technique. By confirming that the experimental results closely match the predicted values, researchers can have confidence in the method’s effectiveness and reproducibility.

Cryoconcentration also demonstrated effectiveness in concentrating individual anthocyanins from the extract obtained with water modified by the eutectic mixture. Compared to the initial concentrations, the final concentrated extract showed substantial increases, with values 11 times higher for delphinidin-3-O-glycoside, 8 times higher for petunidin-3-O-glycoside, and 7 times higher for malvidin-3-O-glycoside. These results highlight the ability of cryoconcentration to enrich the extract with individual anthocyanins, offering the potential for the production of high-quality concentrated extracts suitable for food use. Preservation of pigments and bioactive compounds during concentration is crucial, and cryoconcentration shows promise [21,22].

### 3.5. Green Metrics

Based on the Green Certificate assessment, the recovery of anthocyanin-rich extracts and their cryoconcentrates can be considered green approaches since they showed values above 90. However, due to the lower energy consumption and produced waste, the obtention of the anthocyanin-rich extract is more sustainable than the cryoconcentrates (as shown in Table S1). Similar results are also observed when evaluating the EcoSacale values (as shown in Table S2). According to Van Aken’s classification [36], EcoSacale scores above 75 are considered excellent, scores above 50 are acceptable, and scores below 50 are deemed unacceptable.

Therefore, ultrasound-assisted extraction (UAE) using water modified by a eutectic mixture as the solvent can be categorized as an excellent green approach, while cryoconcentration makes the process merely acceptable. This disparity can be attributed to the additional steps involved in obtaining cryoconcentrates, which result in increased energy consumption, costs, and environmental impact. Furthermore, apart from the residual exhausted plant matrix, cryoconcentrates generate additional waste in the form of ice fractions, contributing to higher E-factor values (as shown in Table S1). Nonetheless, considering the high efficiency of the first cycle and relatively lower waste generation, cryoconcentrate 1 emerges as the more sustainable option among the concentrated extracts.

### 3.6. Response of Anthocyanin Extract to pHs

**Figure 4:**
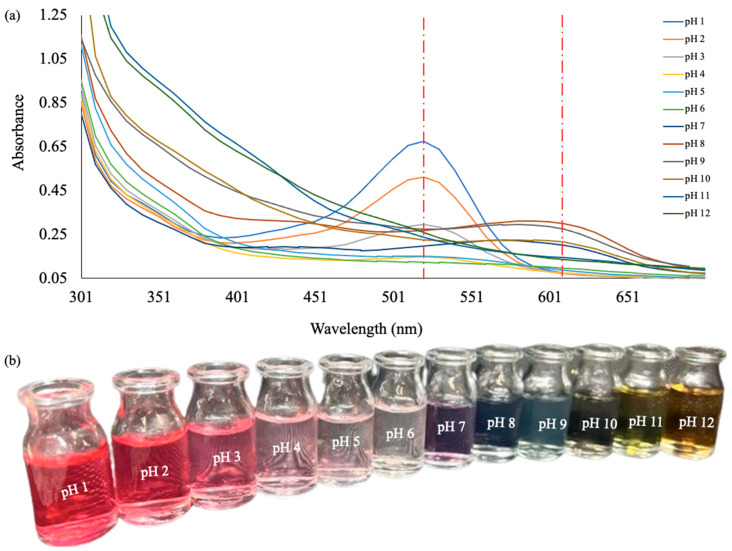
(**a**) Color variations of anthocyanin concentrate extract (**b**) and UV-vis spectra of the anthocyanin concentrate at maximum absorbances of 520 and 610, represented by the dashed line.

Considering the high efficiency of the first cycle and the relatively lower generation of waste, cryoconcentrate 1 was used to analyze the halochromic behavior of anthocyanins. The characterization was performed by UV-visible spectra at different pH values (1–12). Color shifts were observed in concentrated anthocyanin solutions as pH levels increased. The color changed from reddish to light pink (pH 1–6), from purple to blue (pH 7–9), and from light yellow to dark yellow (pH 10–12) (Figure 4b). These color changes stem from pH-induced alterations in the molecular characteristics of anthocyanins, transitioning from the flavilium cation state (pH ≤ 3) to the quinoidal base form (pH 6–7) and then, to the chalcone form (pH ≥ 8) [10,58].

The maximum absorbance in the UV-visible spectra was around 520 nm under acidic conditions. As the pH increased from 1 to 6, the maximum absorbance gradually decreased. With the pH increasing from 7.0 to 12, the maximum absorption shifted to approximately 610 nm (Figure 4a). Similar results were found for anthocyanins extracted from black rice bran using conventional methods (agitation for 24 h in dark conditions) with ethanol: 1 M HCl (85:15, *v*/*v*) as solvent [58]. The color variation of the anthocyanin concentrate with pH suggests that these compounds can be used as natural halochromic indicators in food packaging. This is particularly relevant because, when extracted with an eutectic mixture, the anthocyanins retain their colors, being sensitive only to pH and not to light and temperature. Additionally, since anthocyanins are cryoconcentrated, the amount required to be used as indicators for food packaging is much lower than non-concentrated extracts.

## 4. Conclusions

The use of water modified by eutectic mixtures associated with the ultrasound-assisted method (UAE) was efficient in recovering anthocyanin-rich extract from broken bean hulls. The optimum conditions that maximize the extraction were 8.2% of the eutectic mixture as the water modifier, a temperature of 66 °C, and an ultrasound power of 420 W. The extract recovered with the eutectic mixture as a water modifier revealed protection to TMA at elevated temperatures and light, compared to using only water as solvent. The concentration of extracts was efficiently performed using centrifugation-assisted cryoconcentration, which increased 11-fold the delphinidin-3-O-glucoside concentration of the initial extract. According to green metrics, the presented approach can be considered a green process. Considering the anthocyanin levels obtained and the process sustainability, the production of the concentrated extract using the first cycle of cryoconcentration is the most advantageous. Thus, the first concentration cycle, which exhibited vibrant colors of anthocyanins, was chosen to analyze the color change at different pH levels. Under acidic conditions, the coloration presented pink tones, while in basic medium, it exhibited yellow tones. Overall, enabling technology using eutectic mixtures as water modifiers followed by cryoconcentration proved to be an efficient approach for use as indicators in food packaging due to the quantity and quality of anthocyanins.

## Figures and Tables

**Table 1:** An experimental dataset was obtained from UAE under different experimental conditions and evaluated in terms of total monomeric anthocyanins (TMA), total phenolic content (TPC), and in vitro antioxidant activity by DPPH and ABTS methods.

Essay	*x*_1_	*x*_2_	*x*_3_	TMA	R^2^	R^2^_adj_	TPC	R^2^	R^2^_adj_	DPPH	R^2^	R^2^_adj_	ABTS	R^2^	R^2^_adj_
1	50	150	9	3.7 ± 0.2	0.95	0.92	73 ± 4	0.81	0.77	389 ± 2	0.87	0.85	707 ± 4	0.94	0.93
2	70	450	5	4.1 ± 0.4			105 ± 1			417 ± 9			966 ± 7		
3	50	150	1	2.8 ± 0.1			47 ± 3			276 ± 6			543 ± 6		
4	30	300	1	1.6 ± 0.1			37 ± 7			253 ± 11			373 ± 3		
5	70	300	9	3.9 ± 0.1			112 ± 10			434 ± 2			893 ± 5		
6	50	300	5	3.7 ± 0.1			74 ± 0.5			334 ± 2			728 ± 8		
7	50	450	1	3.2 ± 0.2			44 ± 6			276 ± 3			671 ± 4		
8	30	300	9	3.1 ± 0.1			49 ± 1			274 ± 7			628 ± 3		
9	50	300	5	3.7 ± 0.2			63 ± 9			340 ± 3			659 ± 3		
10	70	150	5	3.9 ± 0.1			92 ± 5			350 ± 5			863 ± 7		
11	50	300	5	3.7 ± 0.2			83 ± 3			345 ± 12			738 ± 5		
12	70	300	1	3.5 ± 0.3			83 ± 9			328 ± 5			872 ± 12		
13	50	450	9	3.9 ± 0.1			90 ± 8			349 ± 8			801 ± 13		
14	30	450	5	3.0 ± 0.2			55 ± 0.4			255 ± 8			522 ± 8		
15	30	150	5	2.5 ± 0.1			68 ± 6			248 ± 5			480 ± 11		

Note: *x*_1_: temperature; *x*_2_: ultrasound power and *x_3_* concentration of eutectic mixture added in water as a modifier. TMA is expressed in mg of C3GE per gram, where C3GE stands for cyanidin-3-glucoside equivalents. TPC is expressed in mg of GAE per gram, where GAE stands for gallic acid equivalents. DPPH and ABTS are expressed in μmol of TE per gram, where TE stands for Trolox equivalents.

**Table 2:** Effect of temperature on k, t1/2, D, Q10, and thermodynamic parameters for anthocyanin degradation between 60 to 90 °C.

Extract	T (°C)	$k\;({min}^{-1})$	R^2^	$t_{\frac{1}{2}}\;(h)$	D (h)	Q_10_	∆H (kJ mol^−1^)	∆G (kJ mol^−1^)	∆S (J mol^−1^ K^−1^)
8.2% of eutectic mixture added in water	60	0.0004	0.89	28.88	95.94	2.50	84.6	195.2	−332.1
	70	0.001	0.95	11.55	38.38	3.00	84.5	198.6	−332.4
	80	0.003	0.96	3.85	12.79	1.67	84.4	201.2	−330.7
	90	0.005	0.8	2.31	7.68		84.4	205.4	−333.5
Water	60	0.005	0.99	2.31	7.68	1.40	27.3	188.2	−483.3
	70	0.007	0.92	1.65	5.48	1.14	27.2	193.0	−483.4
	80	0.008	0.9	1.44	4.80	1.63	27.1	198.3	−485.0
	90	0.013	0.84	0.89	2.95		27.0	202.6	−483.5

Note: T temperature; k: constant degradation rate.

**Table 3:** Recovered solute, ice, percentage of concentrate and efficiency.

Extract and Cryoconcentration Cycle	Anthocyanins in Concentrate (g.mL^−1^)	Anthocyanins in Ice (g mL^−1^)	Concentrate Percentagem (%)	Efficiency (%)	Wp (g g^−1^)	We (g g^−1^)	RMS (%)
Anthocyanin-rich extract	3.55 ± 0.14	-	-	-	-	-	-
1	6.29 ± 0.10	1.19 ± 0.10	49.07	80.98	0.54	0.51	2.02
2	9.02± 0.58	3.06 ± 0.58	51.69	66.12	0.46	0.48	1.72
3	9.29 ± 0.42	5.43± 0.42	85.83	41.61	0.07	0.14	5.10

Note: We: experimental value, Wp: predicted value, and RMS: root mean square.

## Data Availability

The original contributions presented in the study are included in the article/Supplementary Materials, further inquiries can be directed to the corresponding authors.

## Supplementary Materials

The following supporting information can be downloaded at: https://www.mdpi.com/article/10.3390/foods13091374/s1, Table S1: Penalty points to calculate the Green Certificate and E-factor value for anthocyanin-rich extract and its cryoconcentrate from broken black bean hulls; Table S2: Penalty points to calculate the EcoScale for anthocyanin-rich extract and its cryoconcentrate from broken black bean hulls.
