# Supplementary material for: Efficient Anthocyanin Recovery from Black Bean Hulls Using Eutectic Mixtures: A Sustainable Approach for Natural Dye Development
Source: Foods. 2024 Apr 29;13(9):1374. doi: 10.3390/foods13091374 (PMC11083087; doi:10.3390/foods13091374)
Supplement: Supplementary file 1 [file foods-13-01374-s001.zip › foods-2973102-SI.pdf]

Supplementary Materials

Table S1. Penalty points to calculate the Green Certificate and E-factor value for anthocyanin-rich extract and its cryoconcentrate from broken black bean hulls.

Product	Solvent					Energy		Waste	Total PPs	*Green Certificate*	E-factor
	Type	Quantity (mL)	PP_Q	PP_H	Subtotal (PP)	Consumption (kWh)	PP_E	PP_W			
Anthocyanin-rich extract	Water and DES	15	1.41	1	1.41	0.09	1	0.37	2.79	97.21	268.16
Cryoconcentrate 1	Water and DES	15	1.41	1	1.41	3.72	3	0.92	5.34	94.66	1432.30
Cryoconcentrate 2	Water and DES	15	1.41	1	1.41	7.36	4	1.27	6.69	93.31	3477.33
Cryoconcentrate 3	Water and DES	15	1.41	1	1.41	10.99	5	1.36	7.78	92.22	4856.36

PPQ – penalty point for solvent quantity, PPH – penalty point for solvent hazard, PPE – penalty point for energy consumption, PPW - penalty point for waste volume, PPS – penalty points.

Table S2. Penalty points to calculate the EcoScale for anthocyanin-rich extract and its cryoconcentrate from broken black bean hulls.

Product	Solvent	Penalities							*EcoScale*
		Yield*	Costs	Safety	Technical setup	Temperature / time	Workup and purification	Total	
Anthocyanin-rich extract	Water and DES	12.71	0	5	2	2	0	21.71	78.29
Cryoconcentrate 1	Water and DES	12.71	3	5	7	5	1	33.71	66.29
Cryoconcentrate 2	Water and DES	12.71	5	5	7	5	1	35.71	64.29
Cryoconcentrate 3	Water and DES	12.71	5	5	7	5	1	35.71	64.29

Yield – [100-%anthocyanin recovered]/2, being % recovered in relation to maximum anthocyanin reported in the literature (4.8 mg/g) [10].
